# Absence of subtelomeric rearrangements in selected patients with mental retardation as assessed by multiprobe T FISH

**DOI:** 10.1186/1477-5751-11-16

**Published:** 2012-12-21

**Authors:** Suely Rodrigues dos Santos, Dértia Villalba Freire-Maia

**Affiliations:** 1 Department of Genetics and Molecular Biology, Federal University of Rio de Janeiro State (DGBM-UNIRIO), Rio de Janeiro, RJ, Brazil; 2 Department of Morphology and Genetics, Paulista School of Medicine, Federal University of São Paulo (EPM-UNIFESP), São Paulo, SP, Brazil

**Keywords:** Developmental delay, Mental retardation, Subtelomeric rearrangements

## Abstract

**Background:**

Mental retardation (MR) is a heterogeneous condition that affects 2-3% of the general population and is a public health problem in developing countries. Chromosomal abnormalities are an important cause of MR and subtelomeric rearrangements (STR) have been reported in 4-35% of individuals with idiopathic MR or an unexplained developmental delay, depending on the screening tests and patient selection criteria used. Clinical checklists such as that suggested by de Vries et al. have been used to improve the predictive value of subtelomeric screening.

**Findings:**

Fifteen patients (1–20 years old; five females and ten males) with moderate to severe MR from a genetics outpatient clinic of the Gaffrée and Guinle Teaching Hospital (HUGG) of the Federal University of Rio de Janeiro State (UNIRIO) were screened with Multiprobe T FISH after normal high resolution karyotyping. No subtelomeric rearrangements were detected even though the clinical score of the patients ranged from four to seven.

**Conclusion:**

In developing countries, FISH-based techniques such as Multiprobe T FISH are still expensive. Although Multiprobe T FISH is a good tool for detecting STR, in this study it did not detect STR in patients with unexplained MR/developmental delay even though these patients had a marked chromosomal imbalance. Our findings also show that clinical scores are not reliable predictors of STR.

## Background

Chromosomal abnormalities are an important cause of mental retardation (MR) [1]. Most of these abnormalities can be detected by G-banding karyotype analysis, although subtle structural variations are not easily assessed. Most deletions are located near telomeric regions, a common feature in MR patients [2]. Unfortunately, in most patients (60%) the etiology of MR remains unclear [3], despite the use of good quality metaphase preparations in high resolution assays [4].

In subtelomeric regions, the distal segment is shared by many chromosomes while the proximal is larger and shared by a smaller subset of chromosomes [5]. Sequence similarity between non-homologous chromosomes may cause ectopic recombination during meiosis and lead to subtelomeric rearrangements (STRs) [6], with most translocations occurring in these regions [5]. The frequency of STRs in individuals with MR is 4-35% [7], depending on the assays and patient selection criteria used.

Multiprobe T FISH (MT-FISH) [8] is a specific method for detecting STRs in patients with MR who have a normal karyotype; this technique is widely used in this field of investigation and is available commercially. The wide range in the frequency of STR reflects variation in individual responses in this assay, as well as the availability of adequate laboratory infrastructure [9]. These factors must be considered when choosing the appropriate method in studies of STRs.

The primary aim of this study was to assess the usefulness of MT-FISH as a routine diagnostic test for detecting STRs in children and adolescents with early developmental delay, before the onset of MR. A secondary aim was to improve the predictive value of subtelomeric screening in patients selected using the protocol of de Vries et al. [10]. These individuals had evidence strongly suggestive of a chromosomal imbalance, despite a family history of MR.

## Materials and methods

### Patient selection

Patients were recruited from the genetics outpatient clinic of HUGG-UNIRIO. The cohort consisted of 15 patients (five females and ten males; age range: 1–20 years old) with learning disabilities and/or MR who presented developmental delay. These subjects had a variable family history of learning disabilities, developmental delay or MR and a normal karyotype, as assessed with a high resolution assay. Dysmorphic features, congenital defects or malformations were recorded systematically during clinical examination. An unknown cause of learning disabilities, developmental delay or MR, or uncommon features in known syndromes, were considered as inclusion criteria. Two sets of twins with the same clinical presentation and two second-grade male cousins without similar clinical features were included in the sample. Tests for fragile X syndrome were done in all males and, to exclude other possible causes of MR, the patients were screened for inborn errors of metabolism and underwent serology tests for toxoplasmosis, rubella, cytomegalovirus, herpes virus and syphilis [11]. Subsequently, the clinical criteria of de Vries et al. [10] were applied and a score of 3 was chosen as the minimum for selecting patients for MT-FISH. All parents/guardians of the patients signed an informed consent form prior to participation in the study. Ethical approval was obtained from the appropriate hospital committees. (HUGG – CEP:22/2003; HSP/UNIFESP CEP: 1070/08).

### Cytogenetic technique and molecular cytogenetic analysis

#### Cytogenetic analysis

Cytogenetic analyses done in the Cytogenetic Laboratory at UNIRIO were used to exclude known chromosomal abnormalities. Lymphocyte cultures were established and harvested using standard protocols for high resolution assays. The quality of the metaphase preparations for karyotype analysis and FISH was enhanced by using Cytoclear® (Genial Genetics Upton, Wirral, UK), according to the manufacturer’s protocol.

#### Molecular cytogenetics

STRs were detected using Chromoprobe Multiprobe T System^®^ kits (Cytocell Inc., Banbury, UK) with slight modification to the protocol. Hybridization, washing and staining were done according to standard protocols. The test was repeated if more than two probes could not be scored in the multiprobe device. If one or two probes could not be scored then a specific telomeric probe was used to assess the abnormal result. If an alteration was confirmed, FISH was done in the parents to assess whether the abnormality was inherited. Photographic documentation was used only in abnormal cases.

## Results

Table 1 shows the clinical and laboratory findings for the 15 patients. Ten patients had a familial history of MR, eight had an abnormal electroencephalogram, seven had central nervous system abnormalities detected by tomography or magnetic resonance, seven had mild malformations or birth defects, five had abnormal vision and one had dysacusis.

**Table 1:** Clinical data, complementary exams and familial history of patients

**Patients ID**	**MSA**	**COA**	**JOS**	**CCBN**	**MCSF**	**AJSF**	**YPM**	**JPM**	**RBB**	**RoBB**	**YMB**	**WMC**	**JCTFS**	**LBP**	**LAS**
File number	622076	537048	486383	568752	616423	561932	537160	537162	569901	569575	565119	379028	491362	577557	578487
Birthday	10/8/1985	19/9/1988	10/9/1992	19/7/1984	26/9/2004	28/1/2002	20/4/1995	20/4/1995	2/9/1997	2/9/1997	28/4/1992	24/6/1989	2/9/1995	13/10/1998	21/9/2001
Gender	F	F	F	F	F	M	M	M	M	M	M	M	M	M	M
BW (g)	2360	3100	2850	3350	2580	2625	2750	3000	2230	3050	3340	3630	2010	1235	2155
BL (cm)	45	45	48	50	48	47	-	-	-	-	48	48	43	39	-
BHC (cm)	31	-	-	-	32	33	-	-	-	-	-	-	-	31	-
Age (years)	20	17	13	20	11^5/12^	4	10	10	8^2/12^	8^2/12^	13	15	9	6	4
W (kg)	49.5	43.9	67	67.5	29.8	12.5	41.5	39.2	27.5	28	34.4	63.5	23	17	11.3
H (cm)	142	134	156	150	134.5	97.5	137.5	134.5	125	128	137.5	160	121.5	111	100
HC (cm)	50.5	52	52.5	53	51.5	44.5	54	53	51	51	52	58	51	49.5	48
MF	Y	N	N	Y	N	Y	N	N	Y	Y	N	N	N	Y	Y
DD	Y	Y	Y	Y	Y	Y	Y	Y	Y	Y	Y	Y	Y	Y	Y
Dysmorphism	Y	Y	Y	Y	Y	Y	Y	Y	Y	Y	Y	Y	Y	Y	Y
CS	N	N	N	N	N	N	N	N	N	N	Y	Y	N	N	N
FH	N	N	N	N	Y	Y	Y	Y	Y	Y	Y	Y	Y	Y	Y
LD/MR	Y/Y	Y/Y	Y/Y	Y/Y	Y/Y	Y/Y	Y/Y	Y/Y	Y/Y	Y/Y	Y/Y	Y/Y	Y/Y	Y/Y	Y/Y
X fragile	NEG	-	NEG	NEG	-	NEG	NEG	NEG	NEG	NEG	NEG	NEG	NEG	NEG	NEG
NeuroEv	Y	Y	Y	Y	-	Y	Y	Y	Y	Y	-	Y	Y	Y	Y
CCT / MR	ANL	NL	NL	NL	NL	NL	NL	NL	ANL	ANL	ANL	ANL	ANL	NL	ANL
EEG	ANL	-	NL	-	-	ANL	NL	NL	ANL	ANL	ANL	ANL	ANL	ANL	NL
TORCHS	NEG	NEG	NEG	NEG	NEG	NEG	NEG	NEG	NEG	NEG	NEG	NEG	ANL	NEG	NEG
IEM	NEG	NEG	I	NEG	NEG	NEG	I	I	I	I	NEG	NEG	NEG	NEG	I
BERA	NL	NL	NL	NL	NL	-	NL	NL	-	-	NL	NL	ANL	NL	NL
Ophtalmo Ev	NL	NL	NL	NL	NL	NL	ANL	ANL	NL	NL	NL	ANL	ANL	NL	ANL
DeVries	8	5	4	5	7	8	4	5	5	7	6	5	8	8	7

Birth Weight (BW); Birth Length (BL); Birth Head Circumference (BHC); Weight (W); Height (H); Head Circumference (HC); Malformation (MF); Developmental Delay (DD); Consanguinity; (CS) Familial History (FH); Learning Disabilities (LD)/ Mental Retardation (MR); Neurologic Evaluation (NeuroEv); Cranio Computerized Tomography (CCT) or Cranio Magnetic Resonance (CMR); Electroencephalography (EEG); Toxoplasmosis, rubella, citomegalovirus, herpes virus and syphilis sorology (TORCHS); Inborn Erros of Metabolism (IEM); otoacustic potencials evoked evaluation (BERA); Ophtalmo Evaluation (Ophtalmo Ev).

Female (F); Male (M); Yes (Y); No (N); Negative (NEG); Inconclusive (I); Normal (NL); Abnormal (ANL) clinical score (DeVries).

**Figure 1:**
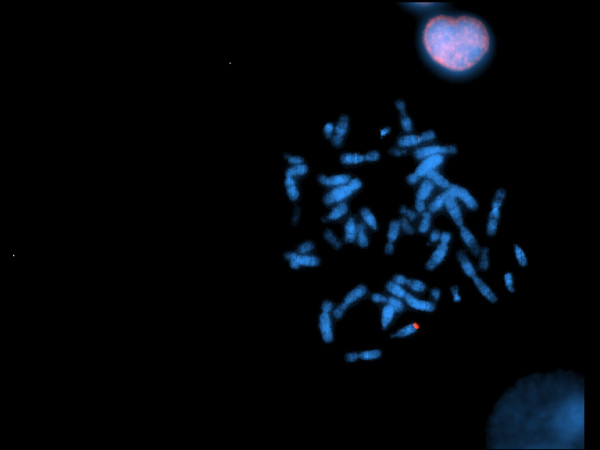
Deletion suspected by MT-FISH.

**Figure 2:**
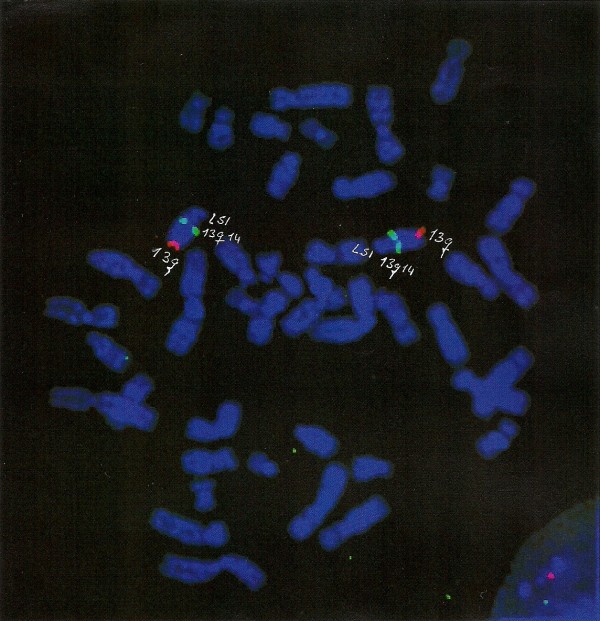
No deletion with 13q subtelomeric probe.

Normal subtelomeric FISH results were observed in 14 cases. A 13q deletion was suspected in one proband of one set of twins (Figure 1), but a specific telomeric probe did not confirm this (Figure 2). The individual concerned was an eight-year-old boy with severe developmental delay who was a compulsive crier when younger and had mild dysmorphic features. He was the first twin born to a young, healthy, non-consanguineous couple with a family history of MR. His brother had similar clinical features but dissimilar results. FISH analysis of their parents revealed no abnormalities. MT-FISH did not detect STRs in any of the patients.

## Discussion

MT-FISH is a precise tool for detecting STRs, but its high cost means that in many developing countries it is first necessary to exclude other more common conditions before choosing this technique. For this reason, we have developed a clinical protocol for investigating the causes of MR [12,13] that can be applied to children suspected of having MR before opting for expensive modern techniques.

Our patients were selected from a large group in which developmental delay was the principal diagnosis and was associated with dysmorphic features and/or malformations. This relationship was initially established in a large screening study in which STRs were found to contribute significantly to unexplained developmental delay and MR with or without a family history of developmental delay or MR [14,15]. In the last decade, several studies using unselected and selected patients have shown that STRs are a prominent cause of MR, especially in idiopathic forms or unexplained developmental delay. Yu et al. [16] reported that the frequency of truly cryptic subtelomeric abnormalities in selected patients was 2.6%. In this study, we examined whether MT-FISH could be used as a routine diagnostic test before the onset of MR. However, this question could not be answered because no STRs were detected in our patients.

Scoring based on clinical criteria has been used in many studies [17-22] to increase the sensitivity of STR detection when MT-FISH is used. De Vries et al. [10] re-examined 29 patients with STRs with regard to their shared characteristics and compared the findings with an MR group without STRs (110 patients). Our group of subjects contained five patients with a low birth weight and nine with a family history of MR. A genetic cause is presumed to underlie half of the cases of undiagnosed patients with idiopathic forms of MR and in many cases there is a family history of MR [12]. Riegel et al. [14] stated that family history was an important selection criterion, especially when MR was associated with dysmorphic features and/or major malformations and growth retardation. As shown here, the number of patients with a low body weight, malformation and a family history was proportionally similar to that reported by de Vries et al. [10]. While the presence of these characteristics and the moderate to severe degree of MR should have increased the likelihood of identifying an STR [10], no STRs were in fact detected. The clinical scores of our patients ranged from four to seven yet no STRs were detected, even though the patients had been carefully selected and were suspected of having a chromosomal anomaly. A similar conclusion was reached by van Karnebeek et al. [23].

Joyce et al. [6] reported one case of STR using MT-FISH that, after a detailed karyotypic review, was found to have semi-cryptic structural anomalies involving a chromosomal imbalance. In the same study, two STRs were found in normal individuals, which suggested polymorphism in subtelomeric regions [24].

A 13q deletion with mosaicism was detected in one of our patients**;** however, there was no phenotypic correlation with the clinical manifestations [25] and FISH with a specific subtelomeric probe was negative. This case indicates the need to establish a clinical correlation with the cytogenetic findings when an STR is detected, especially if the parents or a specific probe are unavailable.

A non-subtelomeric rearrangement could also explain our negative MT-FISH results. In this case, other techniques such as array comparative genomic hybridization may be useful. Based on our findings, we cannot exclude unknown monogenic disease or multi-factorial conditions as at least part of the etiology of MR and developmental delay in these patients.

## Conclusion

In developing countries, there is a need for guidelines for investigating idiopathic MR or unexplained developmental delay in order to establish the best method for a given investigation prior to screening with advanced techniques. MT-FISH is a good tool for investigating the etiology of MR or unexplained developmental delay. However, the cost of this technique in developing countries and the low frequency of STRs mean that the use of MT-FISH may not always be necessary. Finally, clinical criteria were not useful in predicting STRs in this study.

## Competing interests

The authors declare that they have no competing interests with the publication of this work.

## Authors’ contributions

All authors read and approved the final manuscript.

Suely Rodrigues dos Santos was responsible for designing and doing the experiments and Dértia Villalba Freire-Maia provided supervision and general guidance throughout the study. Both authors were responsible for analyzing and interpreting the results and writing the manuscript.
